# Supplementary material for: Southern Indian Ocean Dipole as a trigger for Central Pacific El Niño since the 2000s
Source: Nat Commun. 2022 Nov 15;13:6965. doi: 10.1038/s41467-022-34721-8 (PMC9666355; doi:10.1038/s41467-022-34721-8)
Supplement: Supplementary file 1 — Supplementary Information [file 41467_2022_34721_MOESM1_ESM.pdf]

Supplementary Materials

Southern Indian Ocean Dipole as a trigger for Central Pacific El Niño since the 2000s

Hyun-Su Jo[1], Yoo-Geun Ham*[1], Jong-Seong Kug[2], Tim Li[3,4], Jeong-Hwan Kim[1], Ji-Gwang Kim[1], and Hyerim Kim[5]

[1]*Department of Oceanography, Chonnam National University, Gwangju, South Korea*
[2]*School of Environmental Science and Engineering, Pohang University of Science and Technology, Pohang, South Korea*
[3]*International Pacific Research Center and Department of Atmospheric Sciences, School of Ocean and Earth Science and Technology, University of Hawaii at Manoa, Honolulu, Hawaii, USA*
[4]*Key Laboratory of Meteorological Disaster, Ministry of Education (KLME)/Joint International Research Laboratory of Climate and Environmental Change (ILCEC)/Collaborative Innovation Center on Forecast and Evaluation of Meteorological Disasters (CIC-FEMD), Nanjing University of Information Science and Technology, Nanjing, China*
[5]*Department of Marine Sciences and Convergent Technology, Hanyang University, ERICA, Ansan, South Korea*

Corresponding Authors: Prof. Yoo-Geun Ham, Chonnam National University, Gwangju, Republic of Korea (E-mail: ygham@chonnam.ac.kr)

Table S1. The dominant mixed layer heat budget terms averaged from the developing to mature phase (i.e., June(+1) to January(+2)) of composited El Niño events in the Niño4 region (160°–150° W, 5° S–5° N) with and without the preceding SIOD events during 1998-2019. Left to right observed temperature tendency, zonal advective feedback, thermocline feedback, and Ekman feedback term, respectively (unit: $K \cdot m^{-1}$). Numbers in red parenthesis refer to significant lower and upper bounds at the 95% confidence level.

**Niño4 region (1998-2019)**	**Temperature tendency**	**Zonal advective feedback**	**Thermocline feedback**	**Ekman feedback**
	$\frac{\partial T}{\partial t}$	$-u'\frac{\partial \bar{T}}{\partial x}$	$-\bar{w}\frac{\partial T'}{\partial z}$	$-w'\frac{\partial \bar{T}}{\partial z}$
SIOD + El Niño (percentage)	**0.088**	**0.042** (47.7%) (−0.031 ~ +0.053)	**0.001** (1.1%) (−0.01 ~ +0.007)	**0.016** (18.1%) (−0.02 ~ +0.013)
El Niño only (percentage)	**0.059**	**0.011** (18.6%)	**-0.025**	**0.016** (27.1%)

Table S2. A list of the CGCM and AGCM experiments discussed in the text.

**CGCM**	**Forcing**
Exp_C_CTRL	Seasonally varying climatological SST were nudged in the southern Indian Ocean (65° E – 120° E, 30° – 5° S)
Exp_C_SIOD	Composited SIOD SST anomalies were nudged in the southern Indian Ocean (65° E – 120° E, 30° – 5° S)
Exp_C_EPEN	Composited EP ENSO anomalies were nudged in the tropical Pacific Ocean (120° E – 80° W, 20° S – 20° N)
Exp_C_CPEN	Composited CP ENSO anomalies were nudged in the tropical Pacific Ocean (120° E – 80° W, 20° S – 20° N)
**AGCM**	**Forcing**
Exp_A_CTRL	Seasonally varying climatological SST were prescribed in the southern Indian Ocean (30° E – 130° E, 30° – 0° S)
Exp_A_SIOD	Composited SIOD SST anomalies were prescribed in the southern Indian Ocean (30° E – 130° E, 30° – 0° S)

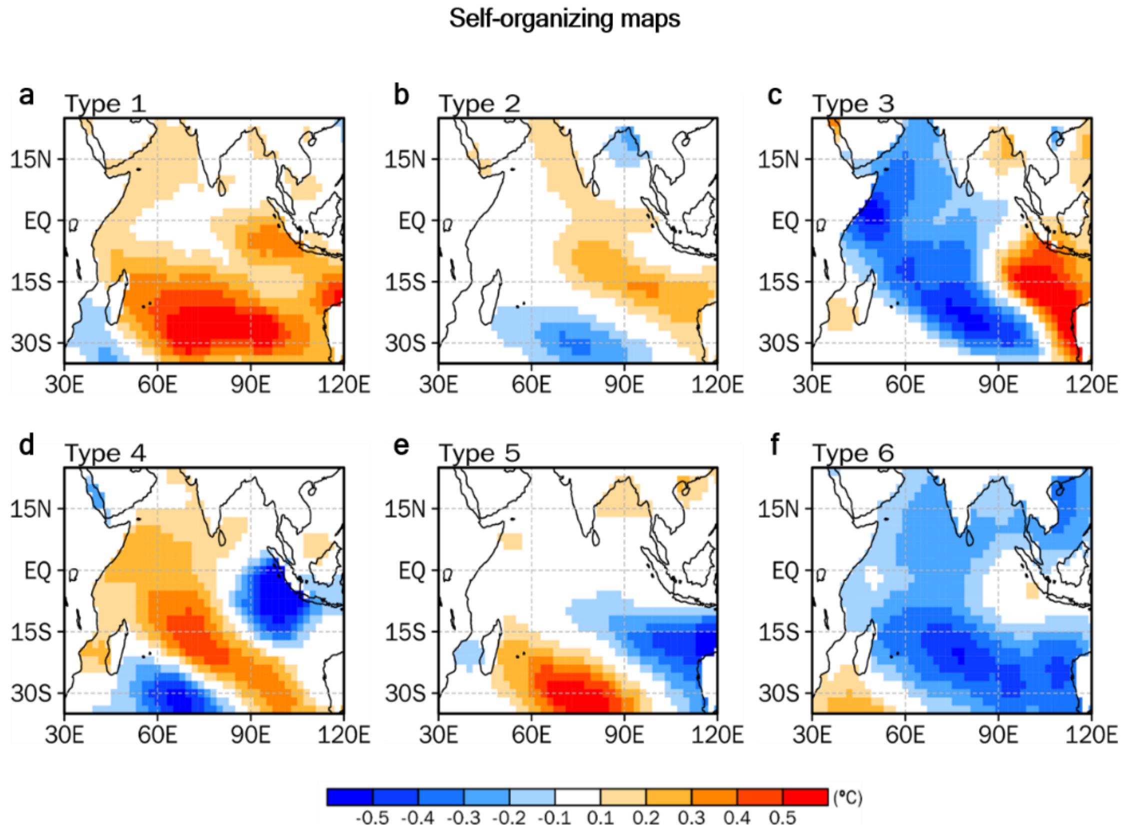

Figure S1. Composite of observed SST (°C) anomalies during the OND season for Types 1-6 from 1976–2019 as revealed by the SOM. A number in the upper left corner on each panel denotes a type index.

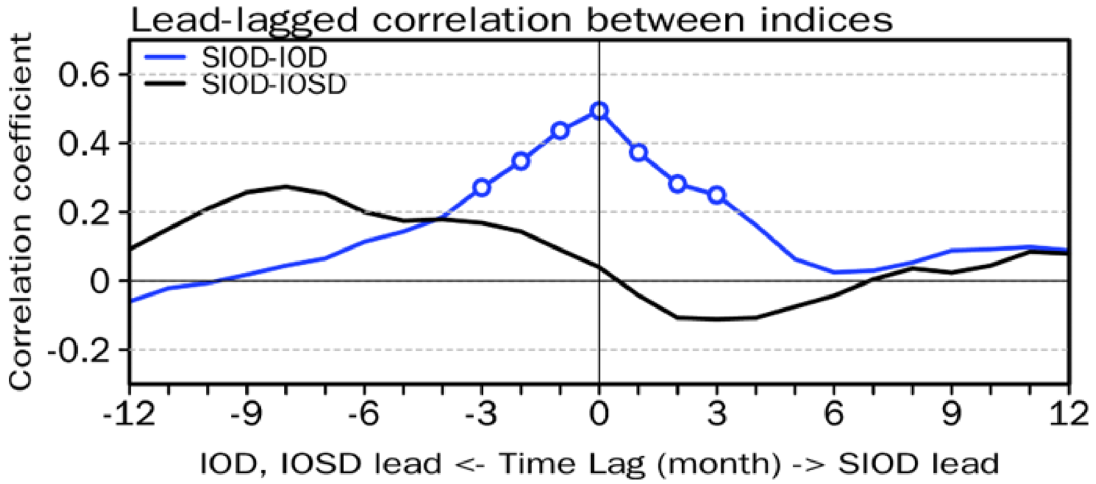

Figure S2. Monthly lead-lagged correlation coefficients between the SIOD–IOD index (blue line), and SIOD–IOSD index (black line) for 1998−2019. Open circles indicate correlation coefficients that are statistically significant at the 95% confidence level.

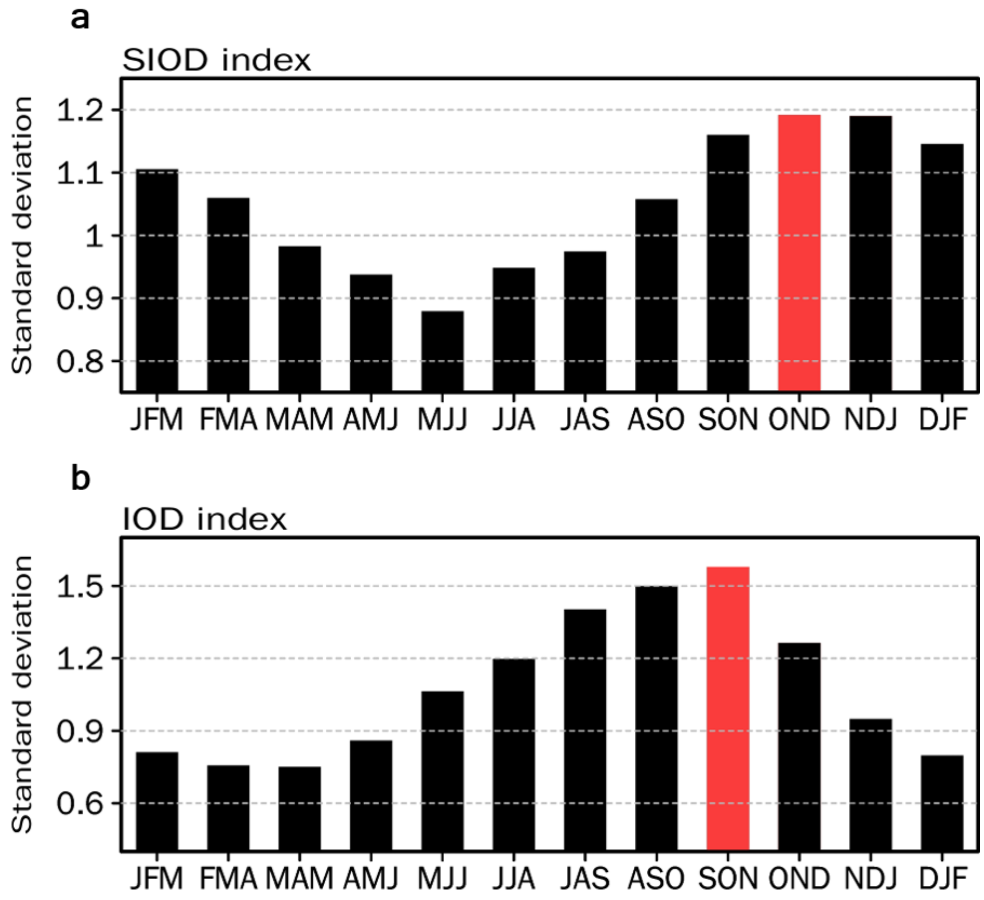

Figure S3. (**a**) The seasonal standard deviation of the SIOD index, and (**b**) IOD index for 1998−2019.

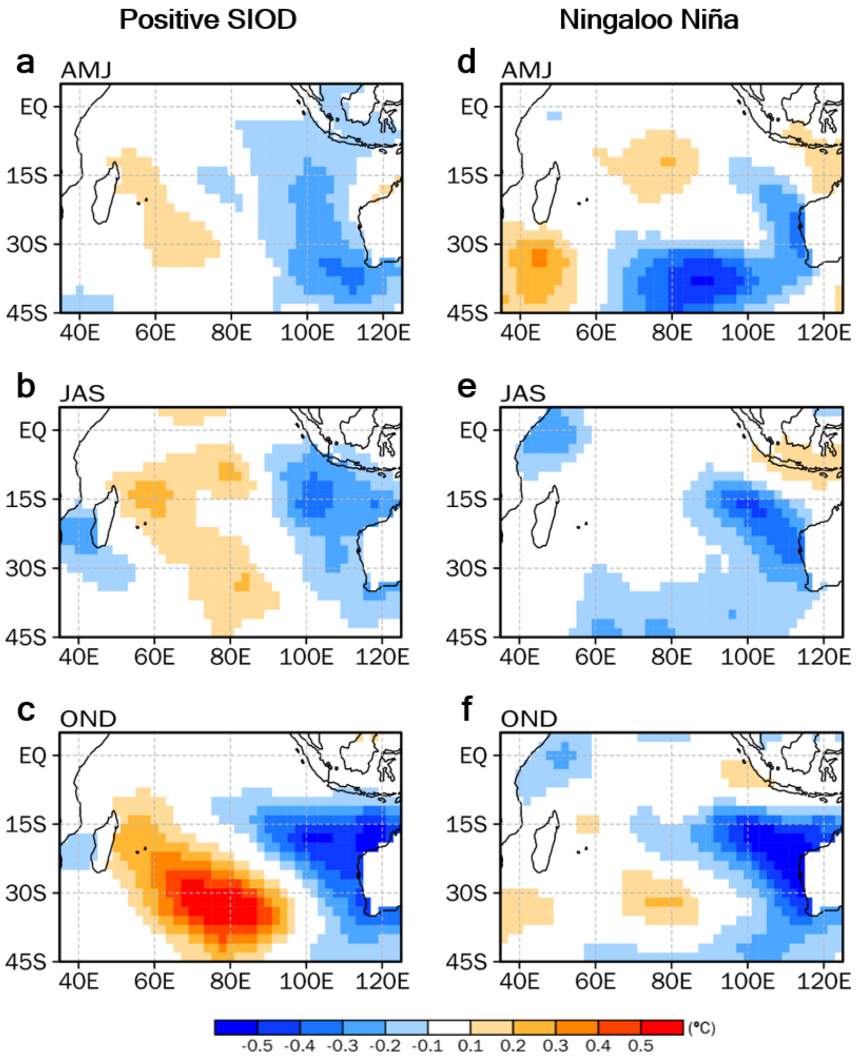

Figure S4. Composite of observed SST (°C) anomalies during the (a) AMJ, (b) JAS, and (c) OND season for positive SIOD events from 1980−2019. (d−f) are identical with (a−c) except for the Ningaloo Niña events.

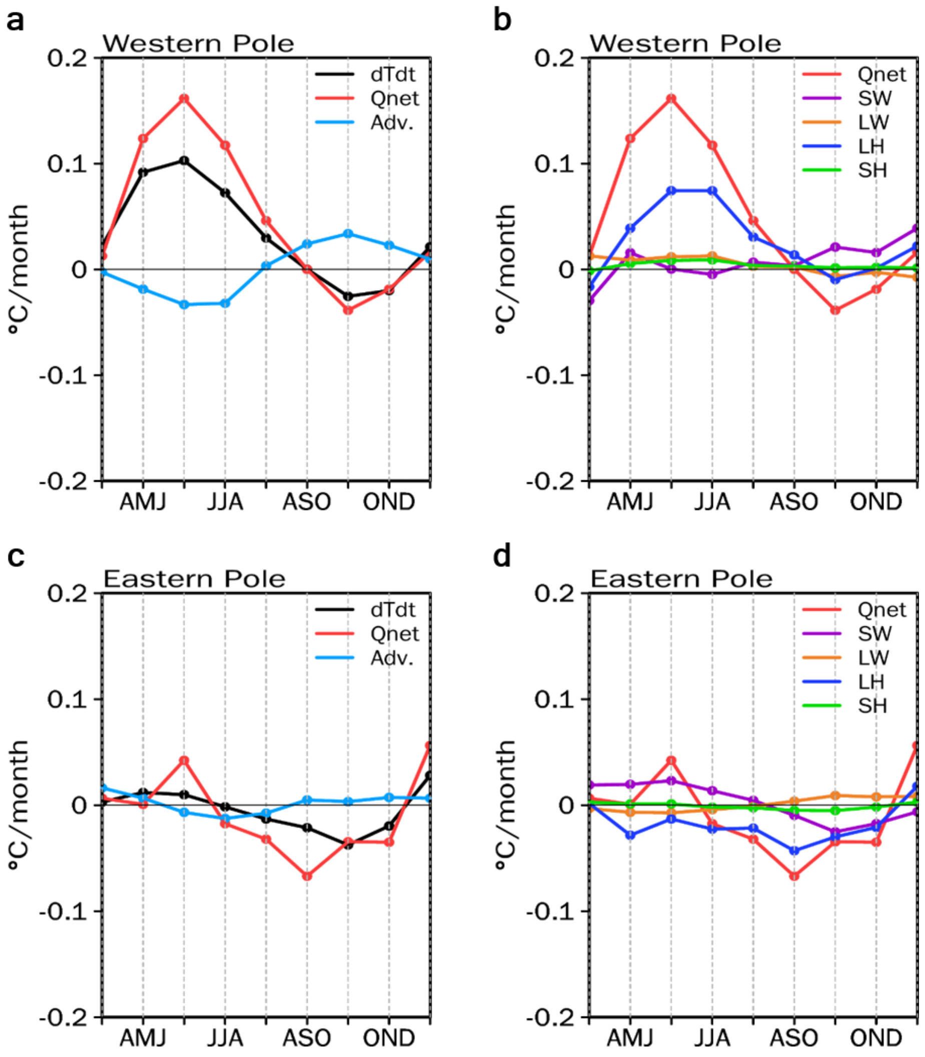

Figure S5. (**a**) Heat budget anomaly of the mixed-layer temperature (left, °C/month), and (**b**) surface heat flux (right, °C/month) anomalies in the western pole (65°–85° E, 25° S–10° S) for positive SIOD events (Type 5) from 1980–2019. In the left panel, black line shows the change rate of the anomalous mixed-layer temperature, red line represents the net surface heat flux anomaly, and blue line indicates the summed oceanic temperature advection terms. In the right panel, red, purple, orange, blue, and green lines correspond to the net surface heat flux (Qnet), shortwave radiation (SW), longwave radiation (LW), latent heat flux (LH), and sensible heat flux (SH) anomalies, respectively. (**c,d**) As in (**a,b**), but for the eastern pole (90°–120° E, 30° S–5° S). Note that a positive sign of the heat flux terms denotes an additional heat flux into the ocean.

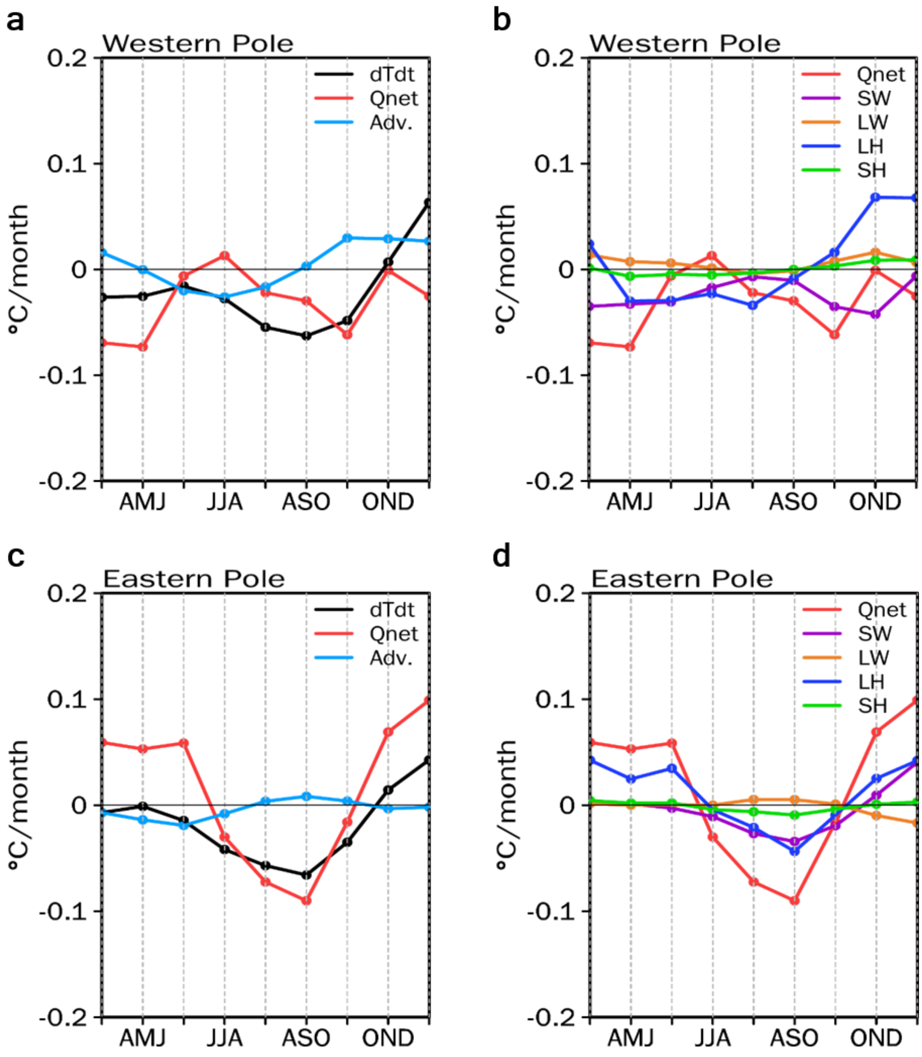

Figure S6. Same as Figure S5 but for Ningaloo Niña events.

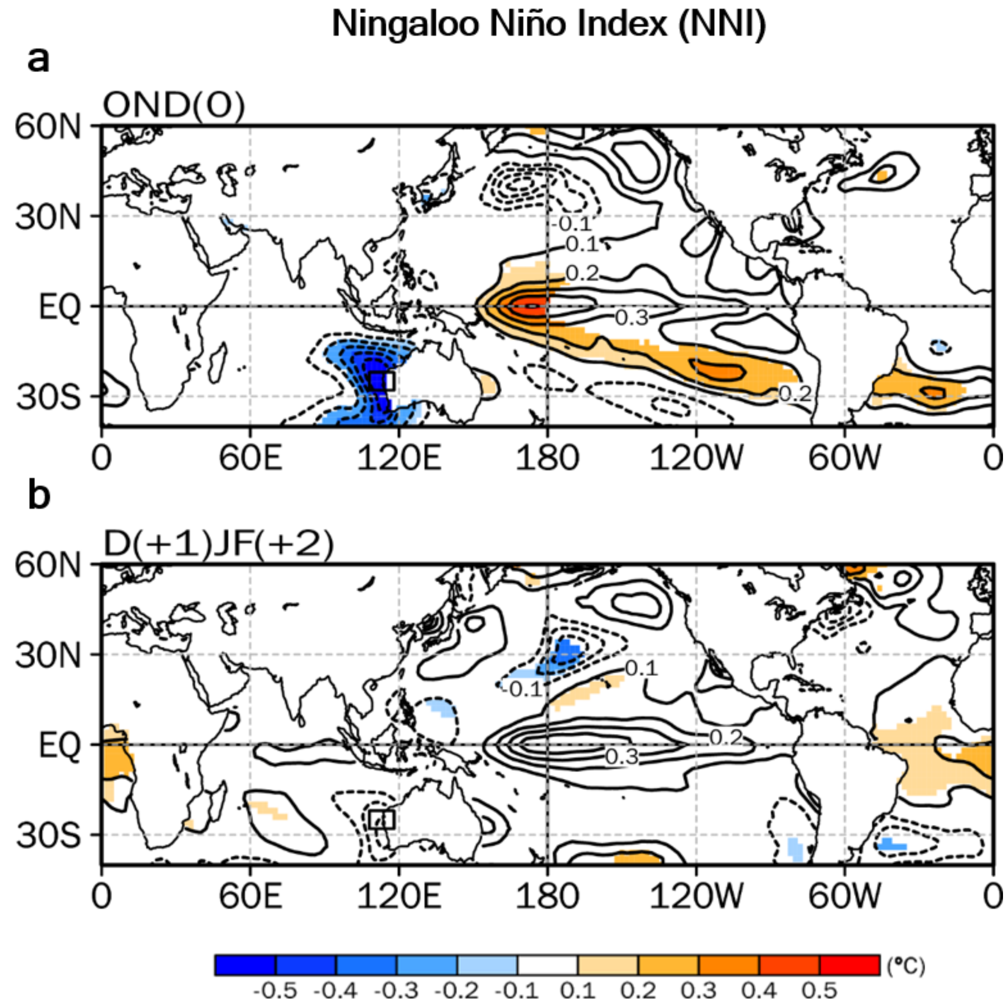

Figure S7. Lag regressions of (**a**) sea surface temperature (SST) (°C) anomalies during the OND season and (**b**) subsequent year's SST (°C) anomalies during the DJF season with respect to the normalized OND Ningaloo Niño index from 1998–2019. We multiplied −1 to the regressed field to match the sign of Ningaloo Niño to the eastern loading of the SIOD event. Shading denotes the region where the statistical significance is above the 95% confidence level.

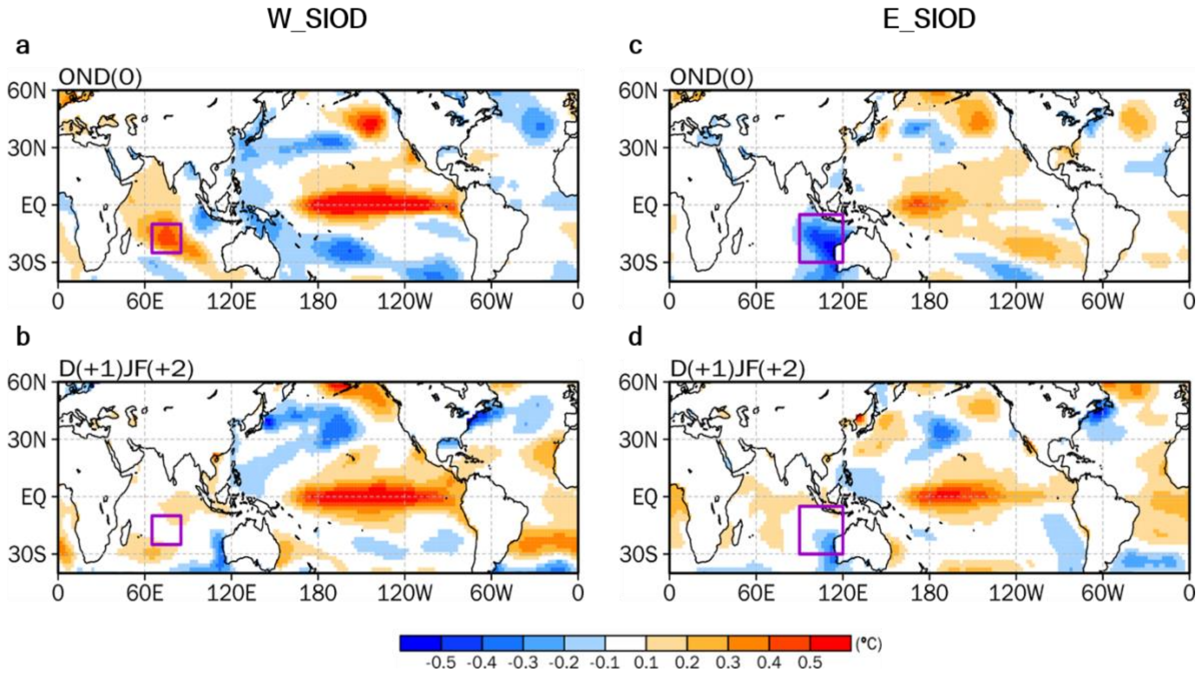

Figure S8. Lagged partial regressions of (**a**) SST (°C) anomalies during the OND and (**b**) subsequent year's SST (°C) anomalies during the DJF season with respect to the western loading of the SIOD after excluding the impact of the eastern loading of the SIOD from 1998−2019. (**c,d**) As in (**a,b**), but for using the eastern loading of SIOD after excluding the impact of the western loading of the SIOD. We multiplied -1 to the regressed fields for the eastern loading of the SIOD to match the sign of Ningaloo Niña event.

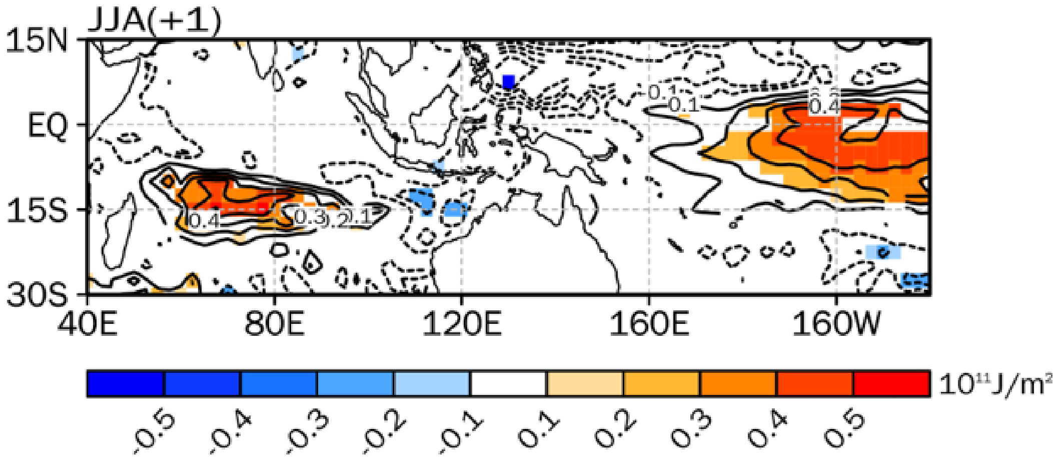

Figure S9. Lag regression of ocean heat content (OHC) (contours, 10^{11} J·m^{-2}) anomalies during the following year's boreal summer with respect to the normalized OND SIOD index from 1998–2019. Shading denotes the region where the statistical significance is above the 95% confidence level.

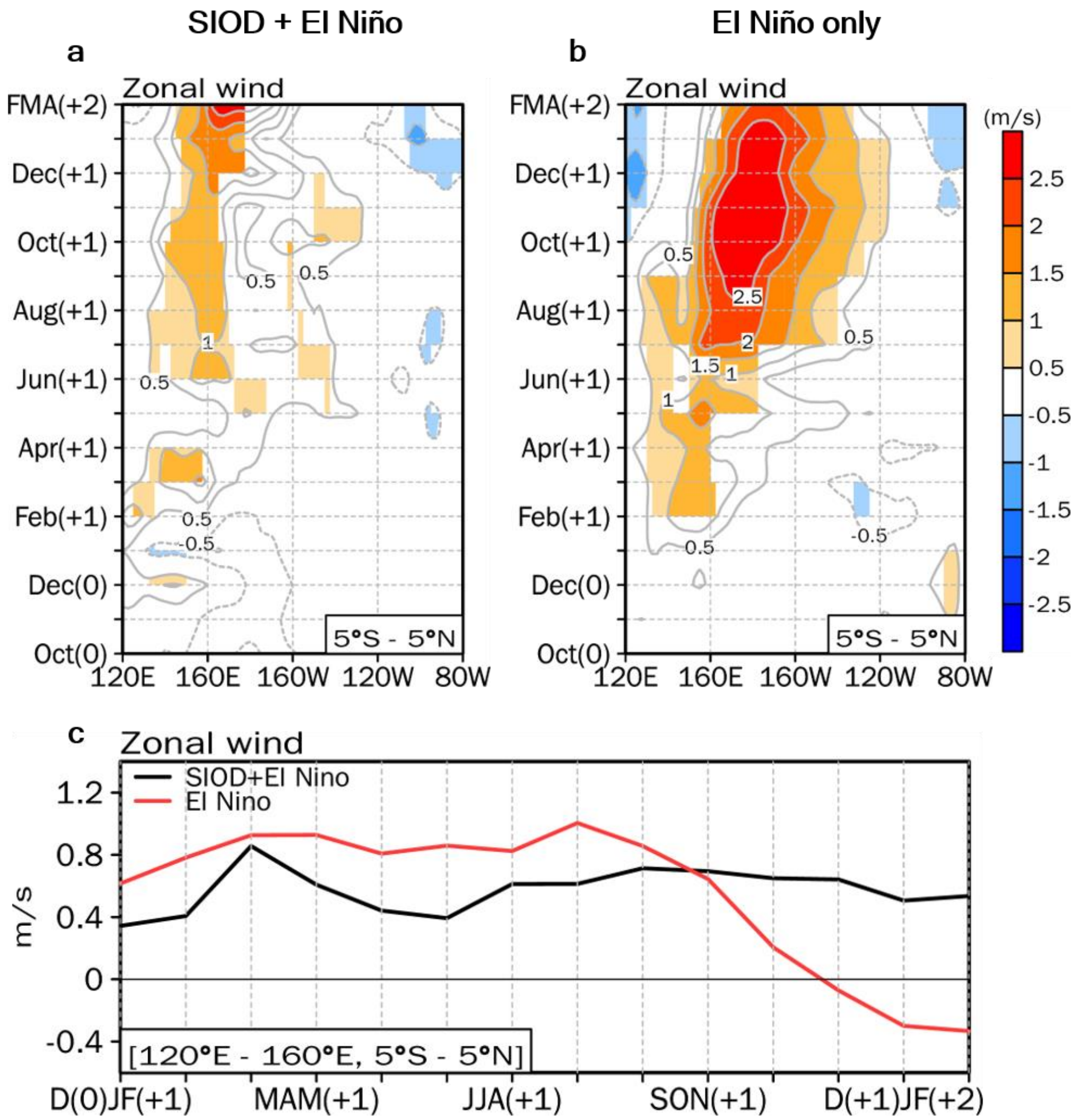

Figure S10. (**a**) Time-longitude diagram of equatorial surface zonal wind anomalies at 925 hPa (colours, $m\cdot s^{-1}$) anomalies for composited El Niño events with the preceding SIOD events from 1976−2019. (**b**) Same as (**a**) except for El Niño events without the preceding SIOD events. (**c**) Time-series of equatorial surface zonal wind at 925 hPa ($m\cdot s^{-1}$) from the D(0)JF(+1) season to the JFM(+2) season for the composited El Niño events with the preceding SIOD (black line) and without the preceding SIOD (red line) events from 1976−2019. The shading in **a,b** denotes the statistical significance at the 95% confidence level.

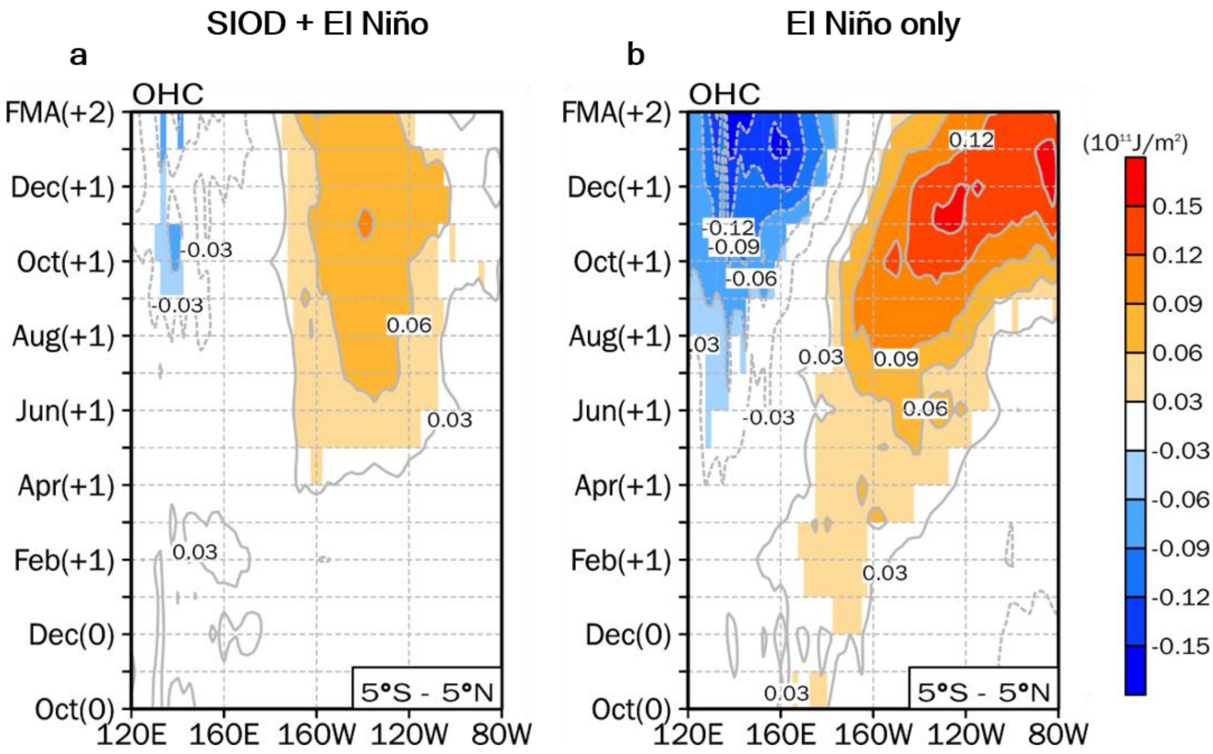

Figure S11. (**a**) Time-longitude diagram of equatorial ocean heat content (OHC) anomalies for composited El Niño events with the preceding SIOD events from 1976–2019. (**b**) Same as (**a**) except for composited El Niño events without the preceding SIOD events. The shading denotes the statistical significance at the 95% confidence level.

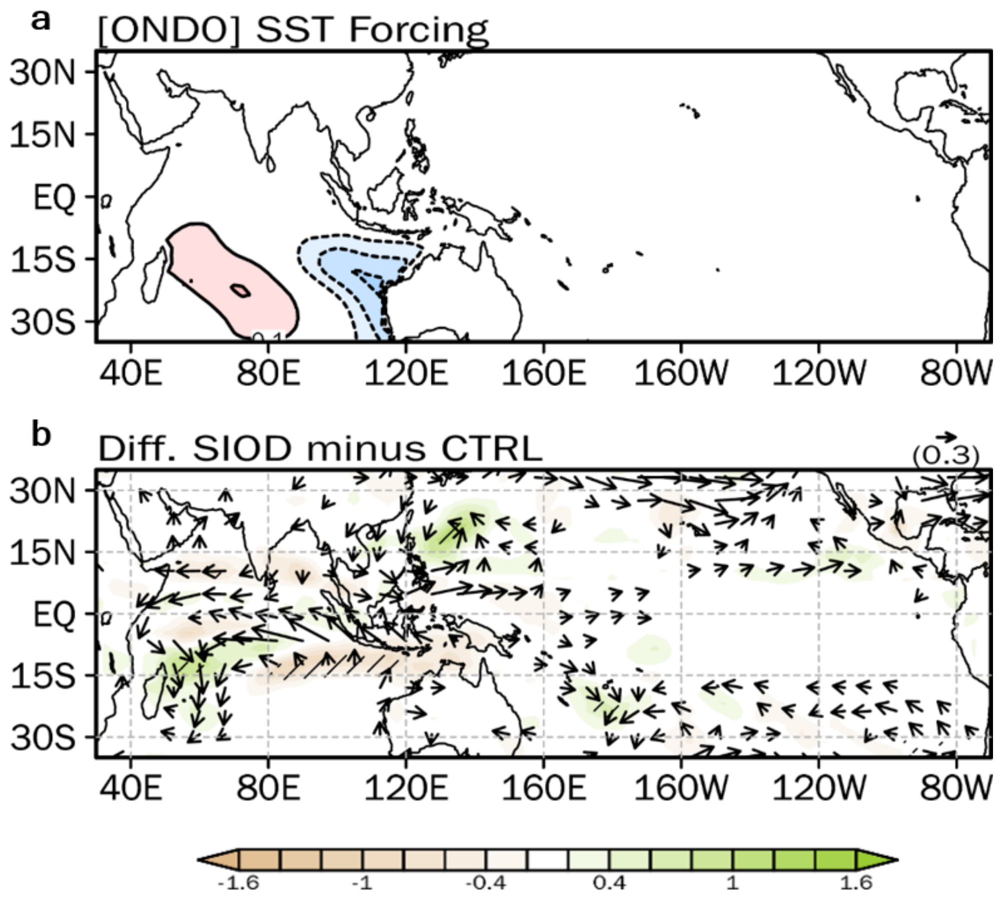

Figure S12. (**a**) SST forcing specified in AGCM experiment. SST forcing is obtained through a regression map of SST (°C) anomalies with respect to the normalized OND SIOD index. (**b**) Changes of precipitation (shading, mm·day^{-1}) and 925 hPa wind (vector, m·s^{-1}) anomalies in Exp_A_SIOD compared to Exp_A_CTRL. The black diagonal lines (vector) denote the regions wherein the statistical significance is above the 95% confidence level.

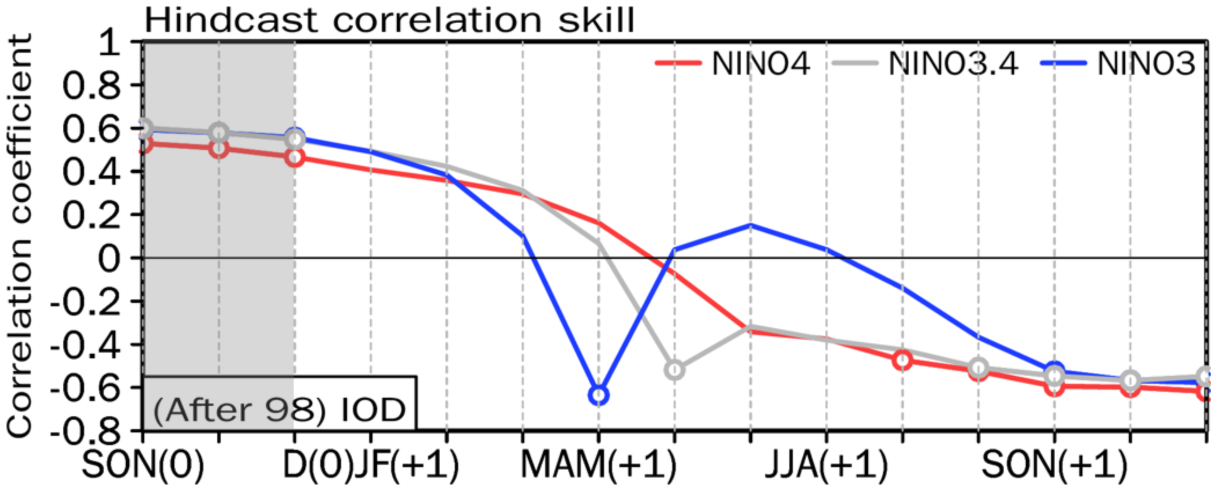

Figure S13. Correlation skills of Niño4 (red), Niño3.4 (grey), and Niño3 (blue) index hindcast from the SON season to the subsequent year's NDJ season using the SON IOD index from 1998–2019. Open circles are statistically significant at the 95% confidence level. A leave-one-year-out cross-validation method was applied.

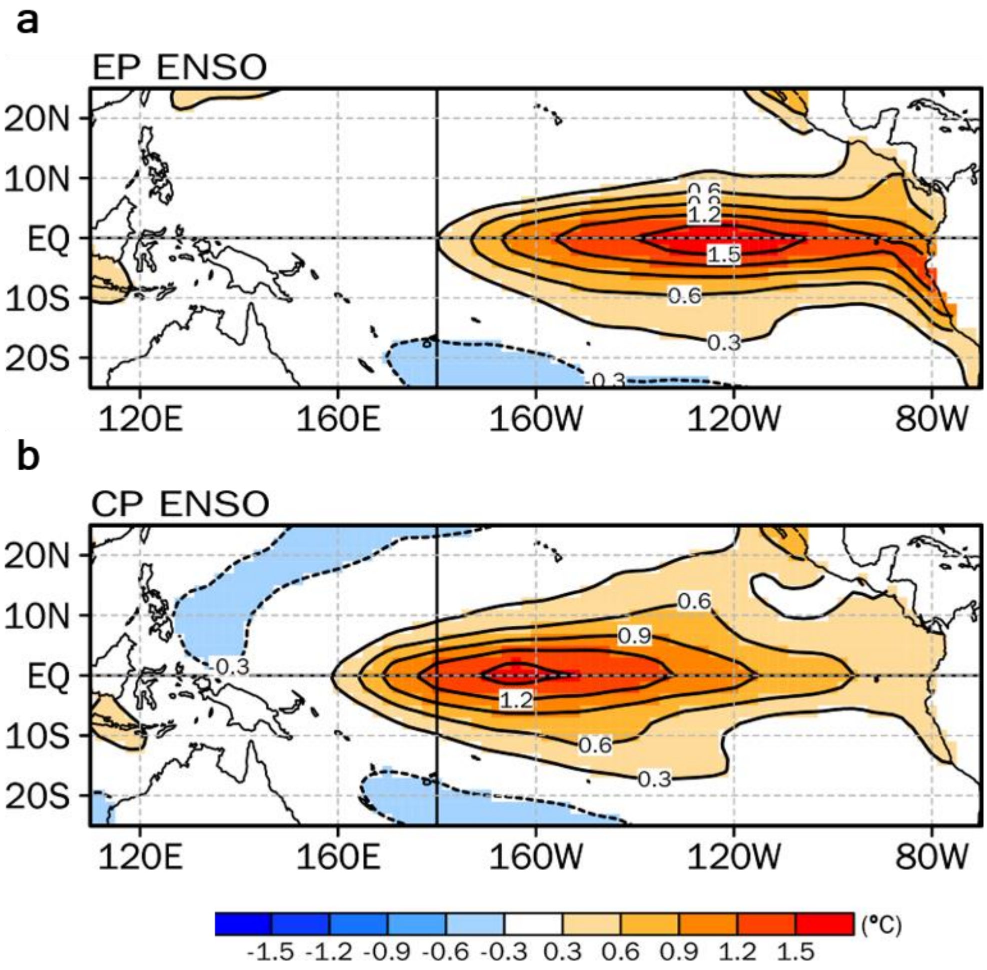

Figure S14. Composite maps of SST (°C) anomalies during the boreal winter for (**a**) EP ENSO and (**b**) CP ENSO events from 1976–2019.

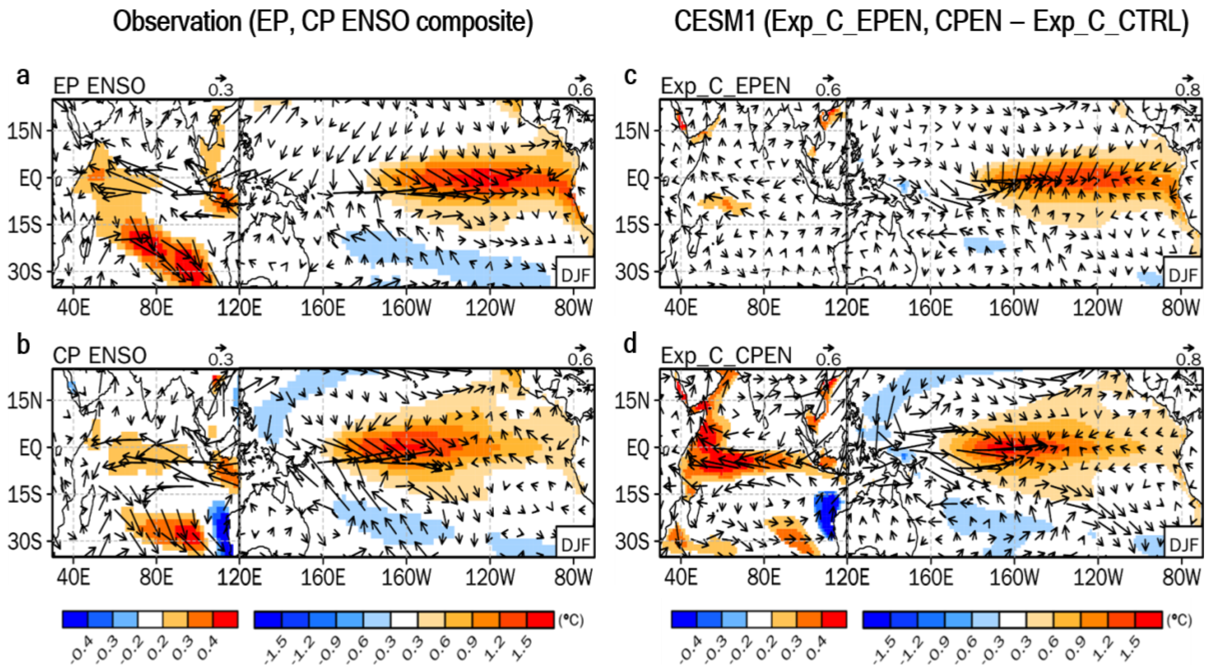

Figure S15. Composite maps of SST (°C) and 925 hPa wind (vector, $m \cdot s^{-1}$) anomalies during the DJF season for (**a**) EP ENSO and (**b**) CP ENSO events during 1976-2019. (**c**) Changes of SST (°C) and 925 hPa wind (vector, $m \cdot s^{-1}$) anomalies in Exp_C_EPEN compared to the control experiment during the DJF season in the CGCM experiments. (**d**) Same as (**a**) except for Exp_C_CPEN. SST forcing is obtained from composited (**a**) EP and (**b**) CP ENSO anomalies.
